# Progesterone modulation of transmembrane helix-helix interactions between the α-subunit of Na/K-ATPase and phospholipid N-methyltransferase in the oocyte plasma membrane

**DOI:** 10.1186/1472-6807-10-12

**Published:** 2010-05-25

**Authors:** Gene A Morrill, Adele B Kostellow, Amir Askari

**Affiliations:** 1 Department of Physiology and Biophysics, Albert Einstein College of Medicine, Bronx, New York 10461 USA; 2 Department of Physiology, Pharmacology, Metabolism and Cardiovascular Sciences, College of Medicine, The University of Toledo, Toledo, OH 43614 USA

## Abstract

**Background:**

Progesterone binding to the surface of the amphibian oocyte initiates the meiotic divisions. Our previous studies with *Rana pipiens *oocytes indicate that progesterone binds to a plasma membrane site within the external loop between the M1 and M2 helices of the α-subunit of Na/K-ATPase, triggering a cascade of lipid second messengers and the release of the block at meiotic prophase. We have characterized this site, using a low affinity ouabain binding isoform of the α1-subunit.

**Results:**

Preparations of isolated plasma membranes from *Rana *oocytes demonstrate that physiological levels of progesterone (or the non-metabolizable progestin R5020) successively activate phosphatidylethanolamine-N-methyltransferase (PE-NMT) and sphingomyelin synthase within seconds. Inhibition of PE-NMT blocks the progesterone induction of meiosis in intact oocytes, whereas its initial product, phosphatidylmonomethylethanolamine (PME), can itself initiate meiosis in the presence of the inhibitor. Published X-ray crystallographic data on Na/K-ATPase, computer-generated 3D projections, heptad repeat analysis and hydrophobic cluster analysis of the transmembrane helices predict that hydrophobic residues L, V, V, I, F and Y of helix M2 of the α1-subunit interact with F, L, G, L, L and F, respectively, of helix M3 of PE-NMT.

**Conclusion:**

We propose that progesterone binding to the first external loop of the α1-subunit facilitates specific helix-helix interactions between integral membrane proteins to up-regulate PE-NMT, and, that successive interactions between two or more integral plasma membrane proteins induce the signaling cascades which result in completion of the meiotic divisions.

## Background

Progesterone induces the meiotic divisions in the amphibian oocyte by activating a signaling system in the plasma membrane (reviewed in [1]). The progesterone receptor on the *Rana pipiens *oocyte surface appears to be localized within the N-terminal external loop of the α1-subunit of Na/K-ATPase [2,3]. The α-subunits of Na/K-ATPase are integral plasma membrane proteins with 10 transmembrane domains and high interspecies sequence homology [4,5]. Based on site-directed mutagenesis data of the rat α1-isoform (reviewed in [3]) and sequence differences between the two ouabain-binding α1 isoforms, we predict that at least seven of the 23 amino acids in the external M1-M2 loop of the low ouabain affinity α1-isoform are involved in progesterone binding [2].

Previous studies in our laboratory indicated that one of the earliest responses to progesterone is a transient increase in phosphatidylethanolamine N-methyl transferase (PE-NMT) [6], an integral plasma membrane protein with 4 transmembrane domains. In the current study, we have examined the kinetics and steroid specificity of PE N-methylation in isolated plasma membranes and find that progesterone (or a non-metabolizable progestin, R5020) binding to the plasma membrane activates PE-NMT within seconds. The initial product of PE-NMT, phosphatidylmonomethylethanolamine (PME), can, alone, initiate meiosis, suggesting that PME is the primary signaling molecule. The fact that both the progesterone receptor (the α1-subunit of the Na/K-ATPase) and PE-NMT are integral membrane enzymes suggests that helix-helix interaction occurs between the M1 and/or M2 transmembrane helix bordering the proposed progesterone binding site [3] and one or more of the 4 transmembrane helixes of the PE-NMT. Each of the 10 helices of the Na/K-ATPase is unique, as well as being highly conserved, both in terms of amino acid sequence, and in 3D structure. Thus, specific helical structures may be critical for the interaction with helices of adjacent membrane proteins.

Computer modeling predicts that only one of the four helices of PE-NMT (M3) is likely to interact with the M1/M2 helices in the α1-subunit of Na/K-ATPase. Structure-function studies indicate that progesterone binding causes the M2 helix of the α1-subunit to rotate, thus facilitating interaction between hydrophobic regions of M2 of the α1-subunit and similar hydrophobic regions of M3 of PE-NMT. The rapid increase in PE N-methylation following progesterone addition indicates that both the α1-subunit of the Na/K-ATPase and PE-NMT are closely associated within the lipid-protein matrix of the oocyte plasma membrane, and are essential components in the steroid rapid response system.

## Results

The emphasis in this study is twofold: 1) an analysis of the kinetics and steroid-specificity of progesterone-induced N-methylation of *in-situ *phosphatidylethanolamine (PE) in isolated, intact oocyte plasma membranes, and 2) the use of computer modeling to predict specific helix-helix interactions between the helices adjacent to the progesterone binding site on the α1-subunit of the Na/K-ATPase and one or more of the 4 helices present in the PE-NMT imbedded in the plasma membrane.

### Progesterone Stimulation of PE-NMT in the intact Isolated Plasma Membrane

**Figure 1:**
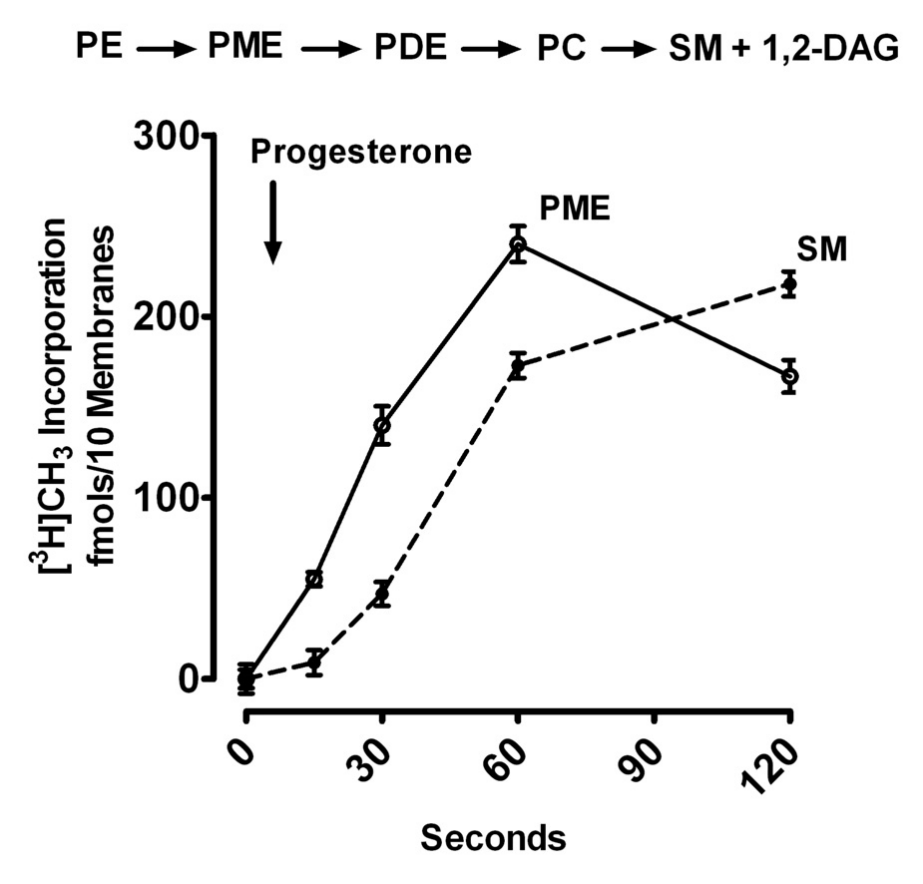
**Time course of [^3^H]Methyl incorporation into PME and SM**. **Upper**: The steps involved in phosphatidylethanolamine (PE) N-methylation and sphingomyelin (SM) synthesis) in *R. Pipiens *oocyte plasma membranes involving PE, phosphatidylmonomethylethanolamine (PME), phosphatidyldimethylethanolamine (PDE), phosphatidylcholine (PC) and sphingomyelin (SM. **Lower**: Net increase in S-adenosyl methionine-derived ^3^H ([^3^H]SAM) incorporation into PME and SM in isolated plasma-vitelline membranes as a function of time after addition of progesterone. The values shown are calculated from ^3^H migrating with phospholipid standards using one-dimensional TLC and are expressed as fmols per 10 membranes corrected for basal levels of the individual phospholipids at the times points indicated. 1,2-DAG (1,2-diacylglycerol) is the product of SM synthase. Values are means ± SEM for oocytes from 3 females.

Figure 1 illustrates the net increase in [^3^H]PME in plasma-vitelline membranes during the first 2 min after addition of 800 nM progesterone to the medium. Isolated membranes were preincubated in Ringer's solution containing [^3^H]S-adenosyl methionine (SAM) for 5 min at 20°C. Progesterone was then added and groups of 10 membranes were collected, rinsed, and extracted at the times indicated (see Methods). Incorporation was expressed as fmols per 10 plasma-vitelline membranes. Following preincubation, control plasma-vitelline membranes contained 2.2 ± 0.04 fmols of [^3^H]PME/10 membranes (mean ± SD, N = 3). Progesterone induced a 20-fold increase in plasma membrane PME within the first 15 sec, increasing 100-fold within 1-2 min. The response was dose-dependent, with maximal PME synthesis at 600-800 nM progesterone. In contrast, only a small increase in N-methylated phospholipids (~30%) was found in untreated (control) membranes over the 2 min period (data not shown). The phospholipid products of N-methylation were analyzed by paper chromatography of the water-soluble bases released after acid hydrolysis, as described by Percy et al. [7]. More than 85% of the radioactivity recovered after acid hydrolysis of PME migrated with monomethylethanolamine standards on paper chromatography.

[^3^H]Sphingomyelin (SM) synthesis also increased 10 - 15 seconds after the initial rise in [^3^H]PME. [^3^H]SM continued to rise as PME synthesis fell, typical of a precursor-product relationship (Figure 1). This is consistent with our earlier finding that, in oocytes prelabeled with [^3^H]palmitic acid, a fall in plasma membrane [^3^H]ceramide coincided with a transient increase in [^3^H]SM [8], indicating that the end product of PE N-methylation (PC) undergoes a transfer reaction with ceramide to form SM and 1,2-diacylglycerol (1,2-DAG).

**Figure 2:**
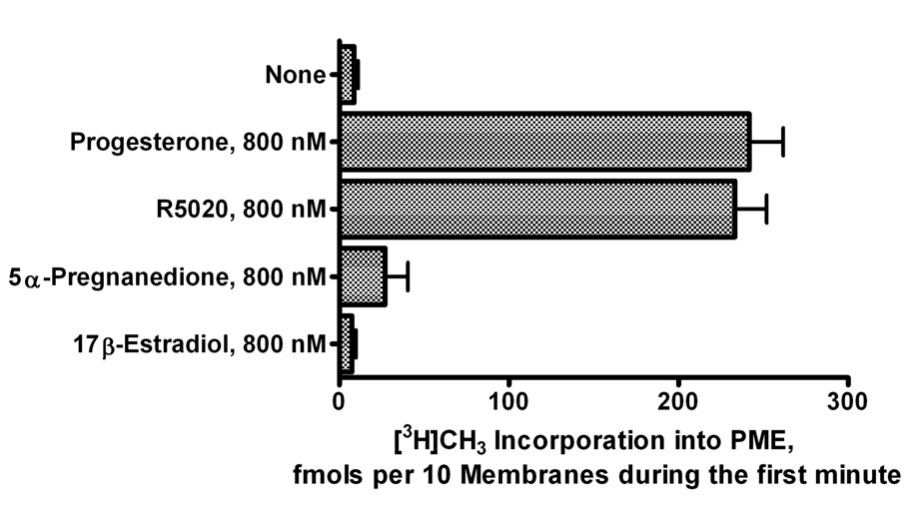
**Induction of PME formation in isolated oocyte plasma membranes membrane by exogenous progesterone, R5020 (a non-metabolizable progestin), 5α-3,20-pregnanedione, and 17β-estradiol**. Plasma-vitelline membranes isolated from denuded prophase-arrested *R. pipiens *oocytes were preincubated with [^3^H]S-adenosyl methionine ([^3^H]SAM) for 2 min at 20°C before addition of 800 nM (final concentration) of the steroid indicated. Individual samples containing 5-6 isolated membranes were frozen in liquid nitrogen at 0, 15, 30, 60 and 120 s after progesterone addition. Membranes were then extracted and analyzed for [^3^H]phosphatidylmonomethylethanolamine (PME) as described in Methods. Values are means ± SEM for oocytes from 3 females.

Figure 2 compares the effects of progesterone, R5020 (a non-metabolizable progesterone analog), 5α-pregnanedione and 17β-estradiol on the induction of PME synthesis during the first 90 seconds after exposure. Progesterone and the non-metabolizable progestin R5020 appear to be about equally effective in stimulating PME formation. 5α-pregnanedione, a major progesterone metabolite formed during the first meiotic division [9], was largely inactive at concentrations as high as 3 μM. Similarly, 17β-estradiol was inactive as a stimulus for PME synthesis (Figure 2) and failed to induce nuclear breakdown in amphibian oocytes [10].

**Figure 3:**
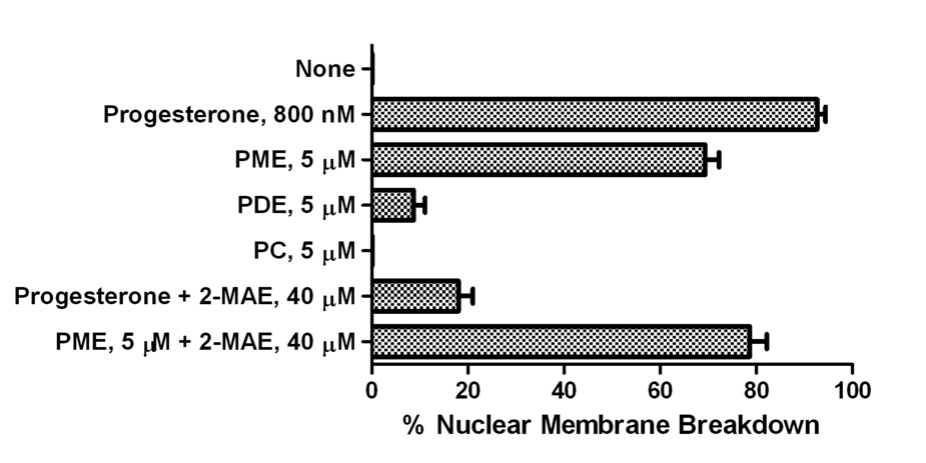
**The induction of meiosis by progesterone compared to its induction by the products of phosphatidylethanolamine N-methylation**. Denuded *R. pipiens *oocytes were transferred to Ringer's solution containing progesterone, PME, PDE and PC and incubated at 20-22°C. These phospholipids, when sonicated in Ringer's solution, formed clear solutions that were stable for several hours at room temperature. Denuded oocytes incubated in the phospholipid-Ringer's micelles for 6 h were rinsed, and then transferred to Ringer's solution for 6 h and nuclear membrane breakdown measured as described in methods. Sibling oocytes were preincubated in an N-methylation inhibitor [2-Methyl(amino)ethane (2-MAE)] for 1 h and transferred to Ringer's solution containing 2-MAE and progesterone or PME. Phospholipids were suspended in Ringer's solution as described in Methods. Results are expressed as means ± SEM for oocyte preparations from 3 females.

We find that PME in micellar form is an effective inducer of oocyte meiotic maturation as indicated in Figure 3. In contrast, PDE has little effect as a meiotic agonist and PC is completely inactive. As also seen in Figure 3, an N-methylation inhibitor, 2-methyl(amino)ethane (2-MAE), inhibits progesterone induction of nuclear membrane breakdown but has no effect on induction of meiosis by PME, indicating that PME acts down-stream in the meiotic events initiated by progesterone.

### Topology and architecture of enzymes (Na/K-ATPase, PE-NMT, SM synthase) involved in the progesterone-response system

Specific interactions between helices are largely driven and stabilized by side chain packing between two or more helices as well as by hydrogen bonding [11]. Crystallographic data [12,13] and computer modeling of individual helices in the α-subunit indicates that each helix of the α-subunit is unique, varying from highly ordered (high helical content) to largely disordered (low helical content). The more ordered helices should have a higher degree of interaction with similarly ordered helices. We have used four computer modeling approaches to: 1) contrast the topology of each protein, 2) compare the 3D structure of the transmembrane helices in the α1-subunit and the PE-NMT, 3) analyze heptad repeats in each transmembrane helix, and 4) unroll, in effect, each helix to allow comparisons of the amino acid patterns and hydrophobic regions.

**Figure 4:**
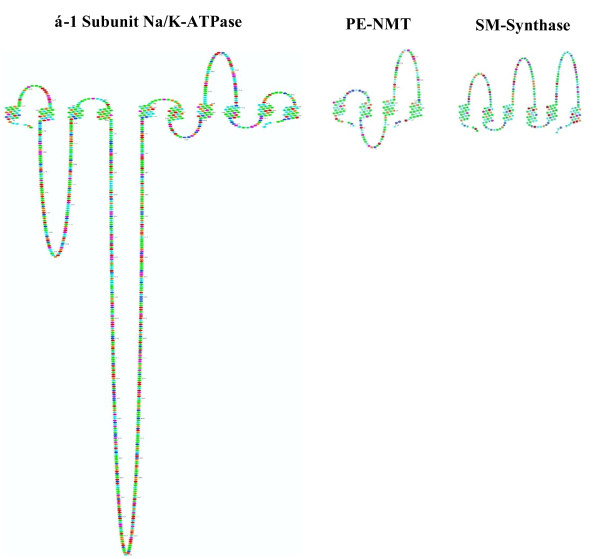
**The topology of the α1-subunit of the Na/K-ATPase (left), PE-N-methyltransferase (center), and SM synthase (right) in Scalable Vector graphics (SVG) format **[14]. The cross section of each enzyme is shown with the lumenal side uppermost and the intracellular environment at the bottom. The N and C termini of all three proteins are located intracellularly.

Figure 4 compares the topology of the α1-subunit of the Na/K-ATPase (rat, Primary accession #Q92123) with PE-N-methyltransferase (rat, Primary accession #Q08388) and SM synthase (rat type 2, plasma membrane, Primary accession #Q4JM44) in Scalable Vector graphics (SVG) format [14]. As outlined in Figure 1, the α1-subunit, PE-NMT and SM synthase participate in the progesterone response. The N and C termini of all three proteins are intracellular. PE-NMT and SM synthase exhibit 4 and 6 transmembrane domains, respectively. The α1 subunit has two large (136 and 434 amino acids) intracellular loops, whereas PE-NMT has a single intracellular loop (198 amino acids). SM synthase has three short intracellular loops.

**Figure 5:**
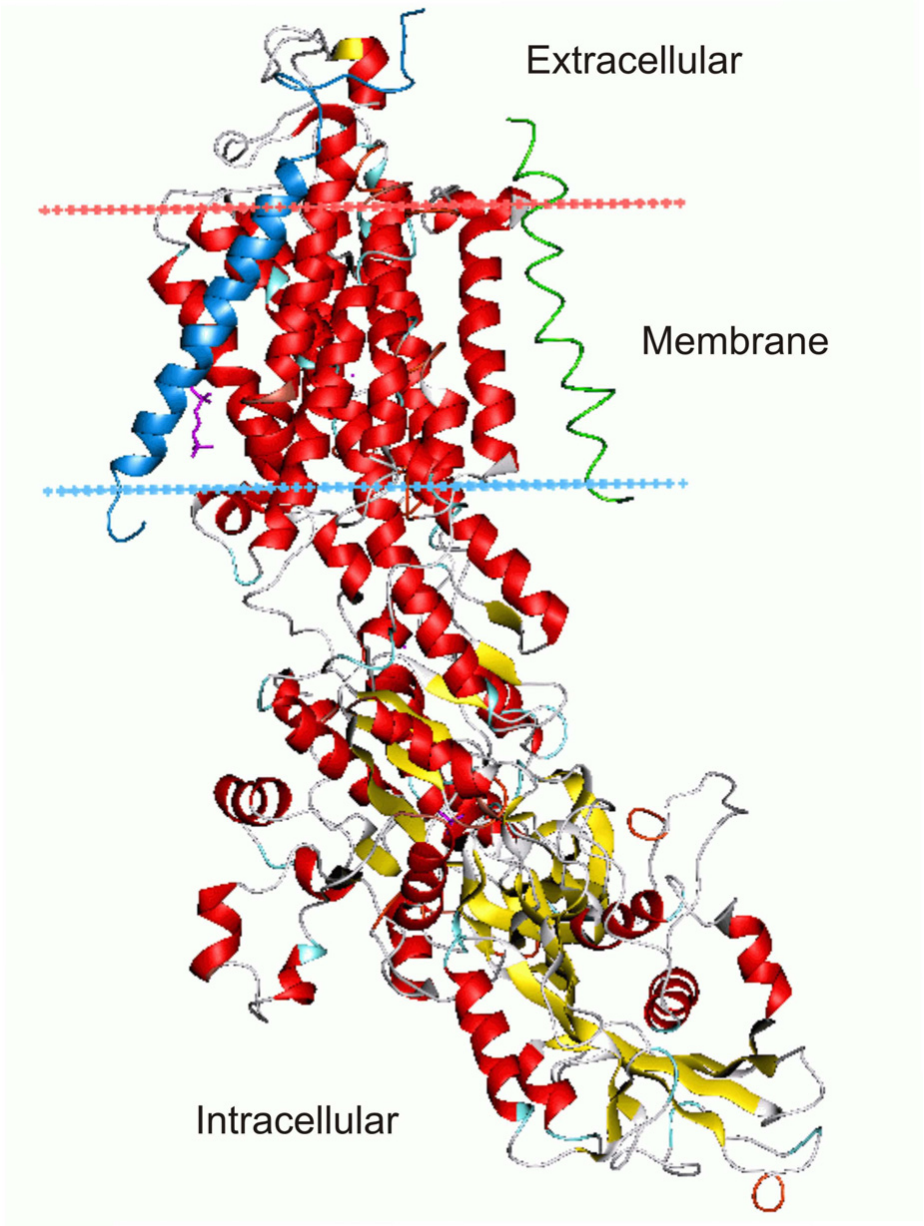
**Architecture of the α1-subunit of the Na/K-ATPase αβγ complex**. α-helices are represented by ribbons and β-strands by arrows with wires that follow the backbone of the α-carbon loops. The β subunit is indicated in blue and the γ subunit in green. The 3D conformation shown is based on the x-ray crystallographic coordinates [12] as analyzed using Pymol Molecular Viewer, ver. 1.1 (DeLano Scientific LLC (pymol.sourceforgr.net). The plasma membrane region is indicated by the red and blue dotted lines with the top as extracellular and bottom as intracellular.

The architecture of the α1-Na/K-ATPase-β γ complex isolated from porcine kidney (Primary Accession #P05024) is illustrated in Figure 5 using published X-ray coordinates (3B8E, Morth et al. [12]). Helices are represented by ribbons and β-strands by yellow arrows. The conformation shown is based on electron density maps of Na/K-ATPase crystals prepared in the presence of Rb^+ ^after solubilization of the membrane-bound enzyme with a non-ionic detergent, and replacement of much of the lipid with detergent. The plasma membrane region containing the helical transmembrane domains is delineated by red (upper) and blue (lower) dotted lines. The β (blue) and γ (green) subunits span the plasma membrane at approximately 45°angles. As seen in Figure 4, helices 2 and 3 are connected by an intracellular loop (136 amino acids), as are helices 4 and 5 (434 amino acids), 6 and 7 (20 amino acids) and 8 and 9 (13 amino acids). Morth et al. [12] pointed out that, in the crystallized form of the enzyme, there is no contact between the helices of the α1-subunit. The only interactions they noted were between the membrane domain of the α1-subunit and the β and γ subunits that are oppositely oriented relative to the membrane plane.

**Figure 6:**
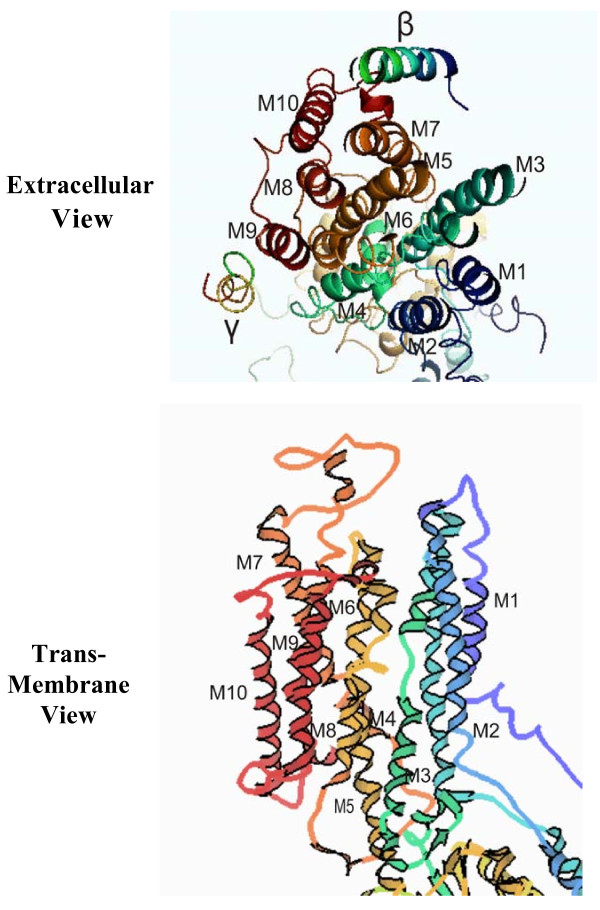
**The 10 transmembrane domains of the Na/K-ATPase α1-subunit seen from the cytoplasmic surface (upper cartoon) and as a transmembrane projection (lower cartoon)**. The x-ray crystallographic coordinates [12] were analyzed using Pymol Molecular Viewer, ver. 1.1 (DeLano Scientific LLC (pymol.sourceforgr.net) and King Display Software, ver. 2.14 (kinnemage.biochem.duke.edu/software/king.php) to generate the upper and upper cartoons, respectively. The relative positions of the lower helices are shown with a 12 ± 2°tilt angle.

Figure 6 compares an expanded representation of the 10 transmembrane helices (numbered 1 through 10) viewed from the cytoplasmic surface (upper cartoon) with a transmembrane projection (lower cartoon) of the X-ray crystallographic coordinates (3B8E) of the α1-subunit of the Na/K-ATPase. The extracellular view (upper cartoon) also indicates the positions of the β and γ subunits. Some transmembrane helices appear to be tilted and packed within the helical bundles so that they are only partially exposed to the membrane-bilayer interface. End-on views of the membrane domains in crystallographic projections indicate that helices 4, 5, 6, 7 and 8 are in the interior of the α1-subunit. Helices 1, 2, 3, 9 and 10 are on the periphery, with helices 1 and 2 close together. The progesterone binding site is located in the extracellular loop between M1 and M2 [3].

**Figure 7:**
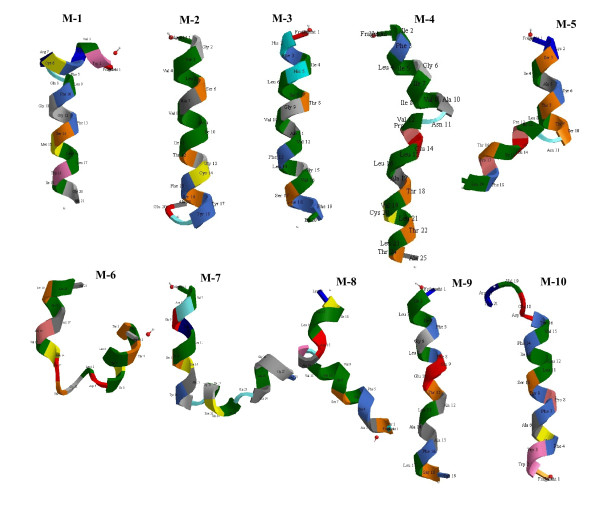
**Comparison of the 3D projections of the 10 transmembrane (M1 - M10) helices of the α1-subunit of the Na, K-ATPase**. Projections are shown as if traversing the plasma membrane from extracellular (top) to intracellular (bottom). Plots were generated using Chem 3D Ultra v. 11.0 (Cambridgesoft.com). Colors indicate individual amino acids.

Comparison of the 3D projections of all 10 helices of the α1-subunit (Figure 7) using Chem 3D Ultra v. 11 (Cambridgesoft, Cambridge Scientific Computing, Cambridge, MA), indicates that M2, M3, M9 and M10 of the α1-subunit are highly ordered, whereas M4, M5, M6, M7 and M8 are largely disordered. M1 has an ordered N-terminal sequence with a hinge-like structure at the lumenal end. The computer-derived 3D projections shown in Figure 7 are consistent with X-ray crystallographic projections derived from purified porcine Na/K-ATPase [12]; the M4 and M6 helices are unwound in the middle and M1 shows a characteristic kink near the lumenal surface. Similarly, M7 is unwound at Gly848, resulting in a kink.

### Analysis of Possible Helix-Helix Interactions between PE-NMT and Na/K-ATPase within the Membrane Bilayer

**Figure 8:**
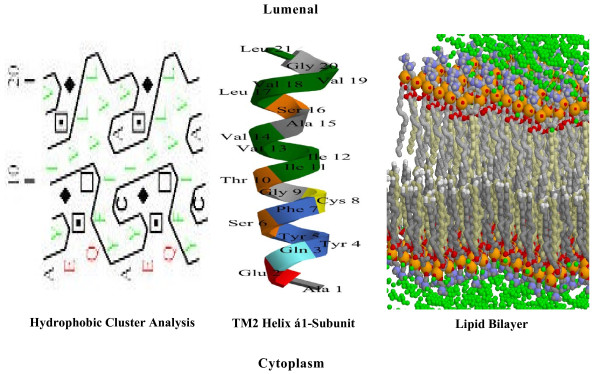
**The 3D projection of the M2 transmembrane helix of the α1-subunit of Na/K-ATPase (center) compared to the corresponding hydrophobic cluster analysis (left) and its relationship to the lipid bilayer of the plasma membrane (right)**. The center projection illustrates the sequence L^134 ^to A^154 ^(Rat Primary accession # PO6685) written in the classical α-helix (3.6 amino acids per turn). The left projection indicates the hydrophobic cluster analysis [15] after unrolling the helix (see text) with the hydrophobic clusters circled. Filled diamonds indicate glycine, squares with a point (serine) or not (threonine), and C for cysteine. The right projection depicts a stylized crystal cartoon of a lipid bilayer containing phosphatidylcholine but no cholesterol. The cartoon is a RasMol image of a phosphatidylcholine bilayer published by E. Martz (see Methods). Green spheres represent water oxygens, blue represent lipid nitrogen, red represent lipid oxygen, and yellow represent lipid phosphorus.

It is useful to visualize the helices both by Chem 3D projections and by computer graphics approaches such as hydrophobic cluster analysis [15] and heptad repeat analysis (reviewed in [16]). Figure 8 compares the Chem 3D projection of the M2 helix of the α1-subunit of the Na/K-ATPase (center) with the hydrophobic cluster analysis (left) of the same transmembrane helix. The top and bottom of the helix represent the lumenal and cytoplasmic surfaces, respectively. A stylized lipid bilayer is illustrated to the right of the M2 helix. Green represents water oxygens, yellow represents phospholipid phosphorus, and red, the fatty acid oxygens associated with the lipid bilayers. The two head group layers, each of which may be 10-15 Å thick, enclose a partially disordered layer of lipid hydrocarbon chains, in which virtually no water molecules are present.

The 3D projection of the M2 helix (middle, Figure 8) is shown as a classical α-helix (3.6 amino acids per turn). Gaboriaud et al. [15] developed an approach that unrolls the helix and makes it possible to visualize the relative positions of both the hydrophobic regions and positions of the shorter side-chains (Gly, Ser, Ala, Val), known to optimize helix-helix packing. The cylinder is shown cut parallel to its axis and unrolled, so that sets of adjacent hydrophobic residues can be encircled. These are termed hydrophobic clusters (left, Figure 8). As some adjacent amino acids are widely separated by the unfolding of the cylinder, the representation is duplicated, making the peptide sequence easier to follow and giving a graphic representation of the microenvironment of individual amino acids. I (Ile), L (Leu), F (Phe) and Y (Tyr) are considered to be hydrophobic amino acids, whereas A (Ala) and C (Cys) are mimetic, i.e. hydrophobic only in a hydrophobic environment. Since P (Pro) lacks a NH group in the peptide backbone and since one of the backbone rotation angles is locked by the proline ring, both the polarity and geometry of the helix are perturbed. Pro often, but not always, induces a helix bend. Consistent with the method of Gaboriaud et al. [15], filled diamonds represent Gly, squares with a point (Ser) or without (Thr), and C for Cys. Pro is denoted by a red star, but is absent in the M2 helix shown. It is apparent that M2 of the α1-subunit has a hydrophobic stripe running parallel to the axis of the helix as well as a horizontal hydrophobic stripe corresponding to the hydrocarbon-rich region of the bilayer.

**Figure 9:**
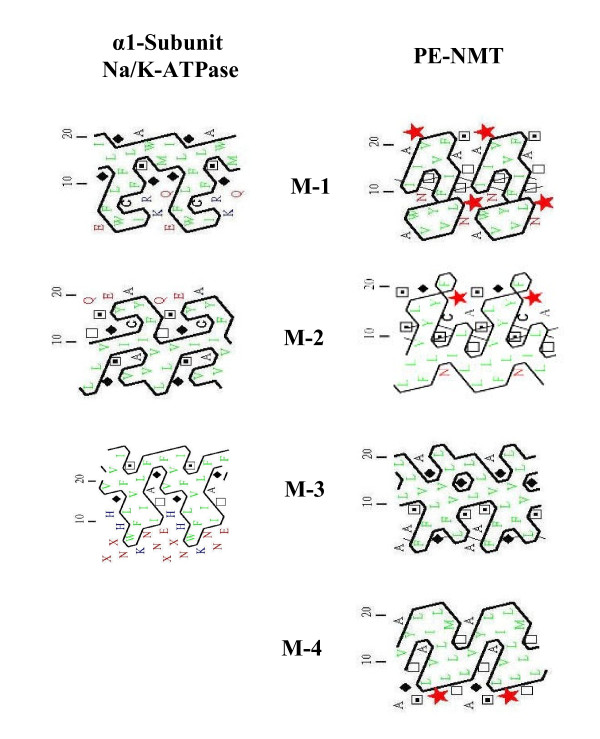
**The hydrophobic cluster analysis of M1 and M2 helices of the α1-subunit of the Na/K-ATPase (left column) compared with a comparable analysis of all four transmembrane helices (M1-M2-M3-M4) of PE-N-methyltransferase (PE-NMT) (right column)**. A red star indicates proline, filled diamonds indicate glycine, squares with a point (serine) or not (threonine), and C for cysteine. Each projection is displayed with the helix unrolled with the top facing the lunenal surface of the cell.

Figure 9 compares the hydrophobic cluster analysis of the three helices closest to the progesterone receptor in the α1-subunit (M1/M2/M3) with a similar analysis of all four helices of PE-NMT. The hydrophobic regions represent potential sites for helix-helix and/or helix-lipid interaction within and between integral membrane proteins. M1, M2 and M3 of the α1-subunit all contain large hydrophobic regions that surround groups of Gly, Ser and Thr; each hydrophobic pattern being unique to a specific helix. Similarly, all four helices of PE-NMT display large, unique hydrophobic regions, but three of the four PE-NMT helices contain proline (red star). Only M3 of PE-NMT contains a highly ordered helix that is more likely to interact with one or more of helices M1, M2, M3 in the α1-subunit.

A third method of visualization of transmembrane domains is by analysis of heptad repeats (e.g. [17]). Table 1 compares the amino acid sequences of the highly-ordered helices M1 and M2 in the α1-subunit closest to the progesterone binding site (columns 1 and 2, respectively), with the one highly-ordered transmembrane helix (M3, column 4) present in PE-NMT. The lumenal surface is at the top, the cytoplasmic surface is at the bottom. Hydrophobic amino acid are in bold, hydrophilic amino acids are italicized. These heptads take the form (abcdefg, column 3, Table 1), where the a and d positions are occupied by hydrophobic residues such as I, L, or V (reviewed in [16]). Folding a sequence with this repeating pattern into an α-helical secondary structure causes the hydrophobic a and d 'repeats' to form an amphipathic strand (see M2 in Figure 8). The hydrophobic 'strand' formed by the M2 helix is apparent in the hydrophobic cluster analysis projection in Figure 8 (the vertical enclosed area containing green hydrophobic residues). Hydrophobic cluster analysis also demonstrates a second hydrophobic strand encircling the helix at right angles to the lumenal-cytoplasm coil in the M2 helix. This horizontal hydrophobic strand corresponds to the central core of hydrocarbon chains in the lipid bilayer (Figure 8) and may thus be involved in helix-lipid interactions.

**Table 1:** The Amino Acid Sequences in the M1 and M2 Transmembrane Helices of the α1-Subunit of the Na/K-ATPase compared with the M3 Helix of the PE-N-methyltransferase

Alpha1-Subunit	Alpha1-Subunit		PE-NMT
TM-1	TM-2	heptad	TM-3
**A**	**L**	A	**F**
**G**	**G**	B	**L**
I	**V**	C	**G**
**W**	**V**	D	**L**
**L**	**L**	E	**A**
**L**	***S***	F	**L**
**M**	**A**	G	**L**
***S***	**V**	A	**G**
**F**	**V**	B	**W**
**G**	**I**	C	**G**
**G**	**I**	D	**L**
**F**	***T***	E	**V**
**L**	**G**	F	**F**
*Q*	**C**	G	**V**
***R***	**F**	A	**L**
**C**	***S***	B	***S***
**F**	**Y**	C	***S***
***K***	**Y**	D	**F**
**V**	***Q***	E	**Y**
**W**	***E***	F	**A**
***E***	**A**	G	**L**

**Figure 10:**
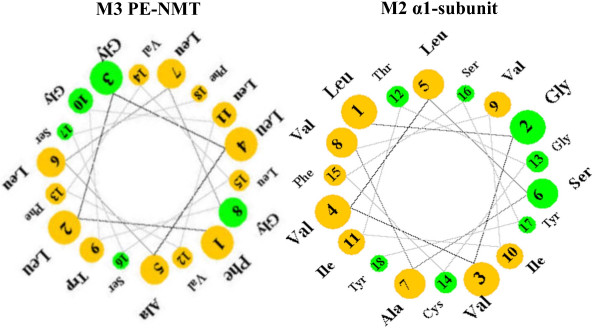
**Helical wheel representations of the amino acid sequences of the M2 helix of the α1-subunit of the Na, K-ATPase and helix M3 of the PE NMT**. The amino acid side chains are projected down the axis of the alpha helix (orthogonal to the plane of the page). The nonpolar residues are in yellow, the polar, uncharged residues are green. As an ideal alpha helix consists of 3.6 residues per complete turn, the angle between two residues is chosen to be 100 degrees and thus there exists a periodicity after five turns and 18 residues. The Figure is a snapshot of a Java Applet written by Edward K. O'Neil and Charles M. Grisham, University of Virginia at Charlottsville, VA (see Methods).

The helical wheel is an additional useful device for visualizing amphipatic helices [18]. Figure 10 compares helical wheel representations of the amino acid sequences of helices M2 and M3 of the α1-subunit of Na/K-ATPase and PE-NMT, respectively. Amino acids in yellow are hydrophobic (nonpolar) whereas those in green are polar, uncharged. The amino acid side chains are projected down the axis of each helix and display periodicity with a repeated unit of length of 7 amino acids. As noted in Table 1 above, the heptad repeat comprises residues a through g, where residue a and d are hydrophobic and define a hydrophobic strand, while electrostatic interactions exist between residues at positions e and g. Helices M2 and M3 are rotated such that residues 1, 4, 8, 11, and 15 in each wheel are facing each other, and constitute the hydrophobic strands of the corresponding helices. Helix M2 of the α1-subunit contains a L, V, V, I and F (Phe) strand, whereas M3 of the N-methyltransferase contains F, L, A, G, L, and L in the opposing hydrophobic strand. Although G at position 8 of helix M3 is indicated as polar, uncharged, it can fit into either hydrophobic or hydrophilic environments, due to its single hydrogen atom side chain.

The most favorable way for two such helices to arrange themselves in an aqueous environment is to wrap the hydrophobic strands against each other, forming a coiled-coil. As shown for PE-NMT M3 (Table 1, column 4), the a and d repeats from lumenal surface to cytoplasm are F, L, G, L, L and F, whereas those for M2 of the α1-subunit are L, V, V, I, F and Y. Residues I, L, F and V have the highest hydropathy indices [19] of the common amino acids and account for 10 of the 12 residues in the M2 and M3 helices. In contrast, the a and d repeats in the M1 helix of the α1-subunit are A, W, S, G, R, and K, with S, R and K being hydrophilic residues (column 1, Table 1). M3 of PE-NMT contains only 2 hydrophilic residues, both near the cytoplasmic interface, whereas M2 of the α1-subunit contains 5 polar residues, 3 of which are at the cytoplasmic interface. The a,d-pattern in M2/M3 predicts that the F-L, L-V, G-V, L-F, and Y-F pairs would interact to form a coiled-coil.

## Discussion

### The progesterone response system in the plasma membrane

The isolated plasma membranes used in these experiments are translucent ghosts, free of other cell organelles [20]. Membrane capacitance measurements indicate that the oocyte plasma membrane surface area is 10-12 times greater than the calculated surface area of a corresponding sphere [21]. This is consistent with freeze-fracture studies that demonstrate the presence of numerous microvilli at the oocyte surface [21]. Based on pulse-labeling and pulse-chase studies, phospholipids in the prophase-arrested oocyte plasma membranes turnover rapidly [1,6] and are recycled to the cell interior with a t_1/2 _of about 15 min [22]. This study examines the potential for cross-talk between two integral membrane proteins, the α1-subunit of the Na/K-ATPase and PE-NMT, in the rapidly recycling lipid bilayer.

Our studies indicate that progesterone binding to an external loop of the α1-subunit of the Na/K-ATPase [3] upregulates an integral membrane protein (PE-NMT) to produce a plasma membrane signaling molecule (PME) (Figures 1 and 2). We have previously shown that [^3^H]glycerol-labeled PME rises within 1-2 min after exposure to progesterone and is derived from the conversion of about 50% of the plasma membrane PE to PME [6]. PE reportedly predominates in the inner lipid bilayer of the plasma membrane, indicating that PME is formed on the cytoplasmic side of the oocyte cortex [23]. A non-metabolizable progestin (R5020) is equally effective in stimulating PME formation, whereas a subsequent progesterone metabolite (5α-pregnanedione) is largely inactive, indicating that progesterone, and not a progesterone metabolite, is the active ligand (Figure 2).

### The lipid-protein microenvironment of plasma membrane

Transmembrane helices are embedded in a lipid bilayer that has distinctive regions, characterized by polar lipid head-groups and a central core of hydrocarbon chains (Figure 8). In nondividing eukaryotic cells, phosphatidylcholine (PC), sphingomyelin (SM) and glycosphingolipids are found primarily in the outer (exoplasmic) lipid leaflet, whereas the cytoplasmic leaflet is generally enriched not only in phosphatidylethanolamine (PE), but also in phosphatidylserine and phosphatidylinositides (reviewed in [23,24]). The inner and outer regions of transmembrane helices of both the α1-subunit and PE-NMT are therefore exposed to different lipid environments. Lipid asymmetry is maintained by the slow translayer movement of lipids [25] as well as by selective lipid transporters (reviewed in [24]). Transporters include P-type ATPases (10 transmembrane domains) which transport lipids inwards from the cytoplasmic bilayer and by ATP-dependent ABC proteins (15 transmembrane domains) which catalyze outward transport. The N-methylation of PE in the inner lipid bilayer, triggered by progesterone, is followed by the rapid and successive conversion of the first product of N-methylation (PME) to PDE and PC, with further conversion to SM. The newly formed PC and/or SM may in turn be transferred from the inner to the outer lipid bilayer by one or more of the plasma membrane ATP-dependent lipid transporters. Thus, progesterone-induced PE N-methylation leads to an extensive and rapid rearrangement within the lipid bilayers of the oocyte plasma membrane.

### Contributions of transmembrane helices and cytoplasmic domains of the α-subunit of the Na/K-ATPase to cellular regulation

The α1-subunit of the Na/K-ATPase contains several large intra- and extracellular domains as well as 10 transmembrane helices (Figure 4). Most structure-function studies of the Na/K-ATPase in other laboratories have been primarily concerned with ouabain binding sites at the cell surface (e.g. [26]) and with the role of specific domains within the large intracellular loops of the α1-subunit (reviewed in [27]). Studies of α1-subunit interaction with other peptides have been mainly limited to the β and γ subunits (reviewed in [26]). However, Xie and Askari [28] found that, in addition to pumping ions, the Na/K-ATPase of cardiac myocytes interacts with neighboring membrane proteins and with the organized cytosolic cascades of signaling proteins (Src kinase, Ras, p42/44) to send messages to intracellular organelles. Tian et al. [29] have recently reported that Src and Na/K-ATPase form a functional signaling complex. This peptide-peptide interaction may involve cytosolic domains, however, since GST pulldown assays indicate that the SH2 and the kinase domains of Src interact with the CD2 and CD3 cytoplasmic domains of the Na/K-ATPase α1 subunit, respectively. More recently, Shinoda et al. [13] have used x-ray crystalographic structure analysis of transmembrane helix-helix interaction between the α, β and γ subunits to explain K^+ ^binding and transport. The progesterone binding studies [2,3] and the studies outlined here indicate that both helix-helix interactions and cytosolic domains are important in cellular regulation.

### Determinants of Helix-helix interaction in the oocyte plasma membrane

Large variations in shape and in tilt angles of the helices relative to the membrane plane are found in both the α1-subunit (Figures 6 and 7) and in PE-NMT (Figure 9). Other investigators have shown that transmembrane helices may be tilted and packed within the helical bundle and thus only partially exposed to the membrane lipid bilayer (reviewed in [30]). Heptad repeats rich in L, I, F, and Y predominate in the highly ordered M2 and M3 helices of the α1-subunit and PE-NMT, respectively (e.g. Table 1). In addition, hydrophobic residues are the most common in the helical region nearest the cell surface, whereas hydrophylic residues are restricted to the region nearest the cytoplasm. These findings indicate that, going from extracellular to intracellular, the helical residues are exposed to successively different microenvironments (aqueous interface, lipid head group, lipid hydrocarbon regions, etc.). Examination of helical wheel projections (Figure 10) predicts that helix M2 of the α1-subunit and M3 of PE-NMT may form a coiled-coil with the a and d positions L, V, V, I, F and Y, interacting with the corresponding residues F, L, G, L, L and F, respectively (going from outside to inside of the plasma membrane). As described in Results, residues I, L, F and V have the highest hydropathy indexes [19] of the common amino acids and account for 8 of the 10 residues predicted to interact. The higher concentration of hydrophylic residues near the cytoplasmic interface suggests that these residues may interact with the polar ethanolamine head group of the PE molecules, known to predominate in the inner lipid leaflet, and may thus play a role in PE N-methylation.

### Progesterone-induced changes in helix-helix interactions in the oocyte plasma membrane

It has been proposed that helix-helix interactions stabilize membrane proteins and, that changes in tight packing as well as interactions between specific residues within and between individual helices are important in signal transduction [e.g. [31]]. Our previous studies demonstrated that progesterone binds to a plasma membrane site within the external loop between the M1 and M2 helices of the α1-subunit of the Na/K-ATPase [3]. As noted above, only M2 has a highly ordered helix capable of forming a coiled-coil and it is rich in hydrophobic residues L, I, V, F and Y. Similarly, analysis of PE-NMT indicates that only one of the four transmembrane helices (M3) is both highly ordered and rich in hydrophobic residues (Figure 9, Table 1).

Since the progesterone-induced reactions alter the lipid composition of the oocyte plasma membrane [1], it is important to consider the consequence of differences in the relative lipophobicity of individual amino acids in helix-helix interactions. Based on the calculated values for the lipid propensities of individual amino acid resides [32], G, A, S and T are lipophobic, in other words, show the least tendency to interact with lipids within the hydrocarbon region of the plasma membrane. V is lipophobic relative to the head group region of the lipid bilayer and lipophilic relative to the hydrophobic region of the lipid bilayer. In contrast, L, I and F (Phe) are lipophilic and show the strongest preference for interacting with lipid. Thus, helices rich in G, A, S, T and/or V would be lipophobic and would preferentially interact with adjacent transmembrane helices having a similar enrichment of G, A, S, T and V. Since L and F are also associated with short interchain helix-helix distances [33], apolar residues such as L and F should stabilize helix-helix structures independent of their lipophobicity. An examination of the sequence data in Table 1 indicates that both M2 of the α1-subunit and M3 of PE-NMT contain the residues optimal for helix-helix interaction.

## Conclusions

Transmembrane helix-helix interactions may have at least two major functions: 1) intramolecular helix-helix interactions would serve to stabilize the tertiary structure of the protein in the membrane bilayer, and 2) helix-helix interactions between two or more integral membrane proteins appear to regulate ligand-initiated response systems. X-Ray crystallography of the α1-subunit of Na/K-ATPase (Figure 6) indicates that 5 of the 10 helices (M4, M5, M6, M7 and M8) lie largely within the core of the α1-subunit, whereas the remaining 5 helices (M1, M2, M3 M9 and M10)) are on the periphery. Computer-generated projections of each helix (Figure 7) indicate that helices within the core are disordered whereas peripheral helices are largely ordered. This suggests that the disordered helices contribute to and/or maintain the tertiary structure of the α1-subunit, whereas the peripheral ordered helices are available for interaction with ordered helices of neighboring integral membrane proteins.

In our model of progesterone binding to the external M1-M2 loop [3], the polar β and the hydrophobic α surfaces of the planar progesterone molecule interact with opposite sides of the 23 amino acid external loop between M1 and M2. Peptide flexibility is maximal near the midway point in the M1-M2 loop, suggesting that folding could occur within the loop, further stabilizing progesterone binding [3]. This would change the relative positions of M1 and M2 and facilitate helix-helix interaction between M2 of the α1-subunit and M3 of PE-NMT, resulting in the observed PME formation within the first few seconds (Figure 2). PE is largely localized to the inner bilayer [23], suggesting that the initial N-methylation step occurs in specific regions of the inner (cytoplasmic) bilayer of the oocyte plasma membrane. One cannot rule out the possibility that an additional peptide intermediate acts to facilitate interaction between helices. For example, the α1-subunit co-isolates with the highly ordered β and γ subunits (Figure 6), and one or both subunits may have an allosteric effect on the progesterone-induced activation of PE-NMT. Subsequent biochemical steps involving other integral membrane proteins (e.g. SM synthase) are also essential for completion of the meiotic divisions [34]. Thus, multiple peptides seem to be associated with the steroid response system. The sequential up-regulation of PE-NMT and SM synthase would produce rapid local changes in N-containing plasma membrane phospholipids (Figure 1), and account for the observed 1,2-DAG transient [8] as well as an increase in SM (Figure 1).

Since a translayer movement of newly formed phosphatidylcholine (PC) from the inner to the outer bilayer occurs in the plasma membrane [24], SM would also increase in the outer bilayer after exposure to progesterone (see Figure 1). These lipid changes would cause the observed progesterone-induced increase in membrane order (decrease in fluidity) [35] and result in decreased protein mobility. Studies in several laboratories suggest that helix rotation is part of the mechanism for signal transduction involving histidine kinases, adenylyl kinases, methyl-accepting chemotaxis proteins and phosphatases (reviewed in [36]). Helix motion could also arise sequentially as a response to progesterone binding to its receptor, the rapid selective turnover of bilayer phospholipids and from the redistribution of newly synthesized phospholipids between the inner and outer bilayers. As shown in Figure 1, PME is rapidly converted to phosphatidylcholine (PC), which is, in turn, converted to SM via SM synthase, an integral membrane protein (Figure 5; 6 transmembrane domains, plasma membrane type 2 form [37]). These three integral enzymes (Na/K-ATPase, PE-NMT and SM synthase) thus interact sequentially to alter the microenvironment of the plasma membrane and to initiate resumption of the meiotic divisions.

## Methods

### Materials: isolation of plasma-vitelline membranes

Fully grown Rana pipiens oocytes, arrested in first meiotic prophase, were stripped of their follicle envelopes and freed from all thecal cells [38]. These oocytes are termed "denuded". Oocyte plasma-vitelline membranes were isolated, one at a time, in 0.24 M sucrose-1.0 mM CaCl_2 _and pooled, as described previously [39]. Nuclear membrane breakdown was detected by dissection of heat-fixed oocytes (5 min in Ringer's solution at 100°C) using a low power binocular microscope. The large (0.5 mm diameter) nucleus (germinal vesicle) is easily seen as an opaque white sphere within the 2 mm diameter oocyte. After breakdown of the nuclear membrane, the residual nucleoplasm appears as a whitish region in the black pigmented animal hemisphere. Phospholipid micelles were prepared by sonication in Ringer's solution for 3 minutes at room temperature. S-Adenosyl-L-[methyl-^3^H]methionine (80 Ci/mmol) and R5020 were obtained from Amersham Corp., Arlington Heights, IL. 2-Methyl(amino)ethane (2-MAE) was obtained from Sigma Chemical Company (St. Louis, MO). Lipids were obtained from Avanti Polar Lipids (Alabaster, AL).

### Progesterone induction of Lipid Turnover

Oocyte plasma-vitelline membranes were obtained from oocytes in prophase arrest, and the solutions prepared immediately before use. Progesterone (Steroloids Inc., Newport, RI) was dissolved in 95% ethanol; with 1.0 μl added per ml of Ringer's solution where indicated. Five-six isolated membranes per sample were preincubated for 5 min in 200 μl aliquots of medium [40 mM Tris (pH 7.5), 5 mM CaCl_2_, 20 mM Mg^2+ ^(acetate), 0.24 M sucrose] containing 2.5 μCi S-Adenosyl-L-[methyl-^3^H]methionine at 20°C (isotope was used without addition of carrier). After preincubation, 20 μl of steroid were added, and the samples collected and frozen in liquid nitrogen at the times indicated. Isotope was also added to membranes at ice bath temperatures, which were immediately frozen in liquid nitrogen as a reagent control. All experiments were carried out in during the normal breeding period (April-May) for *R. Pipiens *and were repeated on oocytes from 3 females.

Each 220 μl sample was homogenized at ice-bath temperatures with a glass-teflon Potter-Elvejm homogenizer in 0.85 ml CHCl_3_:CH_3_OH (1:2 v/v). The homogenates were transferred to 15 ml glass-stoppered centrifuge tubes and successive 0.3 ml aliquots of water and CHCl_3 _were used to rinse the homogenizer. Homogenate and rinse volumes were combined, vortexed for 1 min, and centrifuged at 2,000 × g for 5 min. The upper aqueous and lower lipid-containing phases were separated; the interfacial material washed twice with 0.35 ml volumes of CHCl_3_, and its upper and lower phases combined with the first fractions. The lower CHCl_3 _phase was washed twice with fresh upper phase and taken to dryness under nitrogen. The lipid was then dissolved in CHCl_3 _containing 0.05% BHT and stored at -35°C under N_2_. Sphingomyelin and N-containing phospholipid (including monomethyl, dimethyl and choline phospholipids) were separated on Silica Gel G plates developed either with CHCl_3_/proprionic acid/1-propranol/water (3:2:2:1 v/v) or with the two-dimensional systems of Skidmore and Entemann [40], Gilfillan et al. [41], and/or Katyal and Lombardi [42]. Phospholipids were visualized with iodine vapor, scraped from the plate, and either transferred directly to counting vials containing BCS scintillant (Amersham Corp., Arlington Heights, IL) or eluted with CHCl_3_:CH_3_OH (2:1). Sonication for 1 min facilitated extraction into the scintillant. Recovery of total dpm applied to the thin layer plates was greater than 96%.

### Computer analysis and peptide topology

The UniprotKB/Swiss-Prot/EMBL database http://www.expasy.org/uniprot was the source for the sequence data for the α-subunit isoforms of the Na/K-ATPase, PE N-Methyltransferase (PE-NMT) and the sphingomyelin (SM) synthase obtained from a variety of species. SIB BLAST searches were carried out using the BLAST network service: NCBI BLAST program reference [PMID: 9254694].

A Residue-based Diagram editor (RbDe) Web site maintained by the Department of Physiology and Biophysics, Weill Medical College of Cornell University, New York, NY, was employed to illustrate the topologies of the α1-subunit of the Na/K-ATPase, PE NMT and SM synthase. Chem 3D Ultra v. 11 (Cambridgesoft, Cambridge Scientific Computing, Cambridge, MA) was used to visualize the 3D structure of the transmembrane helices. Hydrophobic cluster analysis [15] of the amino acid patterns within each transmembrane helix used the internet site:bioserv.rpbs.jussieu.fr. Helical wheel representations were visualized using a Java Applet written by Edward K. O'Neil and Charles M. Grisham (University of Virginia in Charlottesville, VA). The Applet is accessible at http://cti.itc.Virginia.EDU/~cmg/Demo/wheel/wheelApp.html.

The X-ray crystallographic projections of the Na/K-ATPase (3B8E) were processed with the PyMOL Molecular Graphics System, Delano Scientific LLC, Palo Alto, CA http://www.pymol.org and the King Display Software, Kinemage v. 2.1 (Biochem.duke.edu/software/king/php). The RasMol image of a phosphatidylcholine bilayer by E. Martz was used as a spatial reference to the 3D projection of transmembrane helices http://www.umass.edu/microbio/rasmol/cutctw.gif.

## Authors' contributions

GAM was responsible for computer modeling and drafted the manuscript. ABK and GAM carried out the biochemical studies and collaborated on the manuscript. AA jointly conceived of the problem with GAM and participated in design and coordination of the study. All authors read and approved of the final manuscript.
